# Continuous Complete Remission in Two Patients with Acute Lymphoblastic Leukemia and Severe Fungal Infection Following Short-Term, Dose-Reduced Chemotherapy

**DOI:** 10.3389/fphar.2021.599552

**Published:** 2021-06-02

**Authors:** Florian Lüke, Dennis C. Harrer, Joachim Hahn, Matthias Grube, Tobias Pukrop, Wolfgang Herr, Albrecht Reichle, Daniel Heudobler

**Affiliations:** Department of Internal Medicine III, Hematology and Oncology, University Hospital of Regensburg, Regensburg, Germany

**Keywords:** biomodulation, anakoinosis, pulmonary mycosis, inflammation, acute lymphoblastic leukaemia

## Abstract

Spontaneous remission in acute lymphoblastic leukemia (ALL) is a rare phenomenon, which typically involves a pattern of feverish or septic disease followed by quick but mostly transient remission. We report on two male patients (46-year-old (pt. 1) and 19-year-old (pt. 2)) with CD20 positive, BCR-ABL negative common B-ALL. Patient 1 had received dexamethasone and cyclophosphamide (1.2 g) as a prephase therapy, followed by rituximab and a cumulative dose of 200 mg daunorubicin combined with 2 mg vincristine as an induction therapy. Patient 2 was treated with a reduced therapy regimen (Vincristine 1 mg, dexamethasone and 80 mg daunorubicin, 12-month mercaptopurine maintenance) due to (alcohol-related) toxic liver failure and pontine myelinolysis. Both patients developed severe septic disease just few days into induction treatment. Patient 1 suffered from pulmonary mycosis, which had to be resected eventually. Histological work-up revealed invasive mucor mycosis. Patient 2 presented with elevated serum aspergillus antigen and radiographic pulmonary lesions, indicative of pulmonary mycosis. In both patients, chemotherapy had to be interrupted and could not be resumed. Both patients recovered under broad antimicrobial, antifungal and prophylactic antiviral therapy and achieved molecular complete remission. At data cut-off remissions had been on-going for 34 months (pt. 1) and 8 years (pt. 2). Short-term, reduced intensity induction chemotherapy accompanied by severe fungal infections was followed by long-lasting continuous complete remissions in ALL. Thus, we hypothesize that infection-associated immunogenic responses may not only prevent early relapse of ALL but could also eradicate minimal residual disease. The effects of combined cytotoxic therapy and severe infection may also be mimicked by biomodulatory treatment strategies aiming at reorganizing pathologically altered cellular signaling networks. This could reduce toxicity and comorbidity in adult patients requiring leukemia treatment. Therefore, these two cases should encourage systematic studies on how leukemia stroma interaction can be harnessed to achieve long lasting control of ALL.

## Introduction

Acute lymphoblastic leukemia (ALL) is an aggressive hematological neoplasia characterized by accumulation of aberrant, immature lymphatic blasts in bone marrow and peripheral blood. Its incidence is about 1.1/100,000 per year with an age peak in children below 5 years and a second smaller peak around 80 years of age. Due to improvements in therapy protocols 5-year overall survival in adults has risen from ∼30% in the years 1997–1999 to 41.1% in the years 2006–2008. Severe complications, such as infections and hemorrhage, are still a cause of significant morbidity and mortality (Sant et al., 2014).

Spontaneous remission (SR) in ALL is a rare phenomenon (Hirshberg and O’Regan; Höres et al., 2018), which typically involves a pattern of feverish or septic disease, followed by quick but mostly transient remission. Nevertheless, the majority of ALL patients do not show leukemia remission upon infections. Generally, “spontaneous” describes a partial or complete disappearance of a malignant disease without any treatment or a treatment, which is not considered adequate for inducing tumor or leukemia response (Everson 1964; Vernon 2018).

A rare, but continuously observed phenomenon, described since the 19th century is the occurrence of infections in the context of systemic tumor regression or remission. The very early pioneer of the theory that infections represent a kind of systemic therapy against cancer was William Bradley Coley, a surgeon who had foreseen the pivotal importance of tumor stroma interactions and “immune response” The surgeons Tilden Everson and Warren Cole reviewed 176 published cases of SR from 1900 to 1960. They also found co-occurring infection as a frequent theme in SR (O'Neill et al., 2009; Vernon 2018).

Clinical data clearly indicate that both inflammation and inflammation control have the capacity to control systemically spread cancer (Walter et al., 2012; Heudobler et al., 2018a). However, the molecular mechanisms, are not fully understood, yet (Cen et al., 2018; Heudobler et al., 2018a; Heudobler et al., 2018b). So far, clinical results indicate infection-associated inflammation is not sufficient for long-term leukemia control. Correspondingly, all spontaneous ALL remissions related to fever or sepsis showed leukemia relapse within a short period of time (Höres et al., 2018).

Here, we describe two cases receiving short-term induction therapy with reduced dose intensity followed by severe fungal infection necessitating discontinuation of any specific anti-leukemic therapy. Long-term continuous remissions could be observed in both cases.

## Patients and Methods

ALL diagnosis was confirmed in both patients by cytomorphology and flow cytometry. Starting with diagnosis, antibiotic (Ciprofloxacin), anti-fungal (Fluconazol) and antiviral (Aciclovir) prophylaxis was administered according to institutional standards. Both patients were treated outside a clinical trial and gave their written, informed consent for publishing their cases. Data were analyzed retrospectively. Clinical outcomes were followed up until 08/2020. This study was approved by the ethics committee of the University of Regensburg (statement No.: 20-2012-104).

## Results

### Patient 1

**FIGURE 1:**
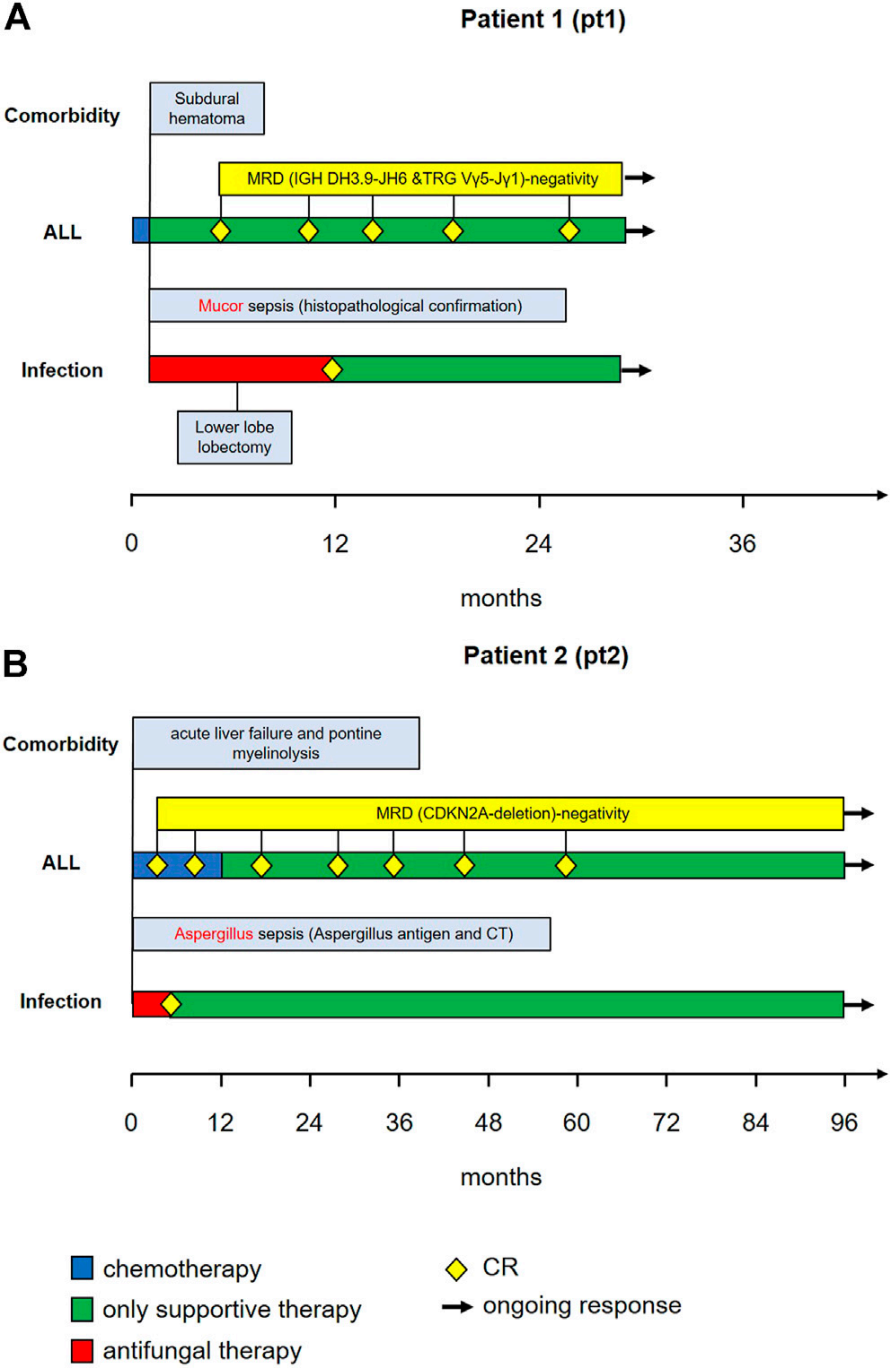
Course of relevant comorbidities (1^st^ row), ALL treatment (2^nd^ row), fungal infection (3^rd^ row) and antimycotic treatment (4^th^ row) in Patient 1 **(panel A)** and Patient 2 **(panel B)**.

**FIGURE 2:**
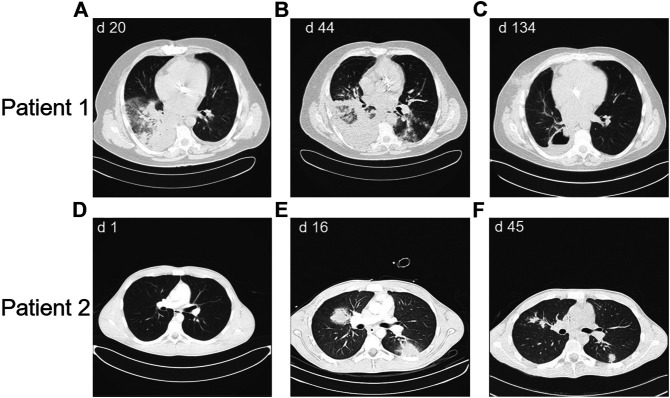
Thoracic CT scans showing the course of mycotic lesions in Patient 1 **(panels A-C)** and patient 2 **(panels D-F)**, d = day after diagnosis of ALL.

Patient 1 was a 46-year-old male with CD-20 positive common B-ALL (Figure 1A).Genetic and molecular-genetic analysis yielded a missing chromosome, 45, XY dic (9;12) (p13;p13) in ALL blasts, plus CDKN2A and 3’ETV6 deletion. He was allocated to the standard risk group of patients according to the GMALL guidelines. Yet, CDKN2 deletion might predict poor prognosis (Xu et al., 2015). Additionally, he suffered from leukemia-associated chronic subdural hematoma. Acute subdural bleeding, complicated by a still measurable activity of phenprocoumon taken because of atrial fibrillation and mechanic aortic valve replacement, was the reason for admission to our university hospital. By then, patient 1 had received dexamethasone and cyclophosphamide (cumulative 1.2 g) as prephase, followed by rituximab, a cumulative dose of 200 mg daunorubicin combined with one dose of 2 mg vincristine. He had also received two intrathecal therapies of 15 mg methotrexate each. Chemotherapy could not be resumed due to active intracranial bleeding. He developed a septic disease pattern eventually leading to intensive care unit (ICU) care because of cardiac decompensation 1 month after admission. A thoracic CT scan showed pronounced pulmonary infiltrates in the lower right lung (Figure 2A), suspicious for fungal infection. Antimicrobial therapy was adjusted empirically. This led to a significant improvement of the patient’s condition and he was transferred back to a normal ward. A dry cough and new pulmonary lesions in the left lung appeared, making further chemotherapy impossible (Figure 2 B). A bronchoalveolar lavage (BAL) revealed mucor mycosis. Since intensified conservative efforts to resolve this infection were unsuccessful, an atypical lung resection became necessary, which removed residual lesions (Figure 2C)

### Patient 2

Patient 2 (Figure 1B) was a 19-year-old male with CD-20 positive common B-ALL. Genetic and molecular-genetic analysis yielded a normal karyotype plus a CDKN2A deletion. He was allocated to the standard risk group according to GMALL guidelines. Similar to patient 1, a non-leukemia-associated severe comorbidity was present at admission. Due to chronic and acute alcohol abuse an acute liver failure with histologically confirmed liver necrosis and accompanying hemorrhagic-necrotizing hepatitis was diagnosed. An initial thoracic CT scan showed no intrapulmonal abnormalities (Figure 2D). Cranial magnetic resonance imaging revealed pontine myelinolysis manifesting with severe neurologic symptoms originating long-term mechanical ventilation starting 3 days after admission. On day 16 after admission, patient 2 developed, a fungal infection of the lung with typical infiltrates in a thoracic CT-scan (Figure 2D), corroborated by elevated aspergillus antigen (Galactomannan) in repeated serological samples. Galactomannan concentrations in serum decreased adequately during broad anti-microbial therapy from 1.6 ng/mL to < 0.5 ng/mL, accompanied by significant improvement of the pulmonary lesions (Figure 2E). In patient 2 the chemotherapy doses were considerably reduced due to the severe alcohol-associated, secondary diseases. Vincristine was administered with 1 mg weekly, thrice combined with dexamethasone and once daunorubicin 60 mg was added, followed by a one year 6-mercaptopurine (6-MP) maintenance. He also received intrathecal therapy with 15 mg methotrexate twice.

In both cases, the severity of comorbidities coupled with the early achievement of molecular complete remission (mCR) prompted, after discussion in an interdisciplinary leukemia board, the discontinuation of intensive leukemia therapy. Only patient 2 received a one-year maintenance therapy, which was stopped due to the slow recovery of the liver function and neurology.

Both patients recovered from fungal pneumonia under broad antimicrobial, while rapidly clearing peripheral leukemic blasts. Whereas patient 2 recovered with continuously size-decreasing residual lesions in both lungs, patient 1 developed one carnifying constant lesion (Figure 2B) of his mucor pneumonia necessitating an atypical resection of this lesion. The additional pulmonary lesions continuously decreased under on-going antimicrobial therapy.

Hematologic remission, paralleled by molecular complete remission (mCR) in the bone marrow samples, was diagnosed after 3 months in patient 1 and after 1 month in patient 2. This means that in both patients, remission occurred after the onset of severe pneumonia, during hematologic recovery phase and after discontinuation of dose-reduced induction treatment.

Molecular CR was measured by the clonal gene-rearrangement of immunoglobulin genes, which were below the detection level (patient 1) and a missing detection of the CDKN2A deletion in patient 2 at first control, respectively. mCR is now on-going for 34 months with 3-monthly controls in patient 1 and 8 years in patient 2 who was regularly followed-up with bone marrow punctures and minimal residual disease (MRD) diagnostics for 5 years.

Thus, short-term, reduced intensity induction chemotherapy paralleled by severe fungal infections was followed by a rapid on-set and long-lasting continuous complete remission of cALL.

## Discussion

Neither short-term, dose-reduced induction therapy nor feverish or septic events are commonly associated with long-term molecular complete remission in ALL.

There is evidence that the immune system can switch from an immunotolerant to an immune activated tumor microenvironment (TME) triggered by chemotherapy and severe fungal infection inducing SR (Diamond and Luhby 1951; Yoruk et al., 2008; Chen and Chuang 2009; Pluchart et al., 2015; Purohit et al., 2015). Possible mechanisms could include inhibition of leukemia re-growth (provoked by the phoenix rising pathway) and control of minimal residual disease (Hoelzer et al., 1996; Seif et al., 2009; Li et al., 2010; Paul et al., 2016). Moreover, pathogen-associated molecular patterns (PAMPs) recognized by toll-like receptor (TLR) 2, TLR3, TLR4, TLR9, and Dectin-1 can be found in aspergillus as well as mucor (McGettrick und O’Neill 2007; O’Neill et al., 2009). Fungal TLR ligands may target both, normal and malignant hematopoiesis (O’Neill et al., 2009; Mansour et al., 2012; Monlish et al., 2016). Since TLRs are widely distributed among different tissues throughout the body, their stimulation next to cytotoxic activity of chemotherapy might be responsible for augmenting antileukemic effects of the TME (Rolf et al., 2015; Heudobler et al., 2019). For example, exposure to beta-glucan released during fungal infections induces expansion of progenitors of the myeloid lineage, which is associated with the recruitment and activation of the innate immune system (Mitroulis et al., 2018). Also unmethylated CpG motifs derived from Aspergillus fumigatus DNA may activate the innate immune system by TLR-9 stimulation (Dalpke et al., 2002; Ramirez-Ortiz et al., 2008). One study using TLR agonists in B-cell precursor ALL cell lines revealed that treatment with TLR2 or TLR9 agonists stimulated allogeneic T cell responses demonstrating an important link between innate and adaptive immunity (Corthals et al., 2006). Moreover, TLR-9 activated plasmocytoid dendritic cells were shown to be a powerful tool for overcoming ALL resistance by NK cell-mediated killing and for reinforcing the graft versus leukemia (GvL) effect following allogeneic hematopoietic stem cell transplantation (HSCT) in an ALL mouse model of residual disease (Díaz-Rodríguez et al., 2017). Targeted stimulation of TLRs and involvement of the innate immune system is associated with enhanced activity of cytotoxic drugs. For instance, treatment of primary ALL samples with the specific TLR2/1 ligand, PAM_3_CSK_4_, triggers caspase-8-mediated apoptosis and sensitizes ALL cells to vincristine-mediated toxicity in vitro (Rolf et al., 2015). Immunization with the vaccine BCR-ABL/GM-CSF/IL-12 and the TLR-9 agonist dSLIM combined with 6-MP induces an innate and adaptive immune response and leads to a very high survival rate in an ALL mouse model (Köchling et al., 2012). Also, in a breast cancer model, intratumoral dendritic cells could enhance efficacy of anthracyclines highlighting the interaction of immune cells and cytotoxic therapy (Vacchelli et al., 2015). In animal models, TLR9 stimulation triggers the break of tumor tolerance and induces immunity against AML and ALL (Hossain et al., 2014; Jo et al., 2020). A phase I trial with the TLR9 agonist (GNKG168) supports the immunomodulatory capacity of a TLR9 agonists in vivo, showing that in children with acute leukemia and present minimal residual disease a unique immune activation pattern can be induced (Ronsley et al., 2019). Taken together, there is a substantial body of evidence, that modulation of the immune system by PAMPs can also induce antineoplastic effects in ALL.

While the two above presented cases show some interesting similarities, there are several limitations to our study. Firstly, we present a very small number of patients that were analyzed retrospectively. Secondly, both patients received different antineoplastic and antiinfectious treatment. Furthermore, we cannot show experimental data on the underlying mechanisms of our findings. Thus, it remains ultimately unclear as to why long-term leukemia control was achieved.

Thus, we need to learn how to create specific (tissue) conditions mimicking those of specific infections that support immune reactions against ALL. While TLR-agonists show poor monoactivity, combining them with e.g. cytotoxic drugs or cancer vaccines might prime the immune system beyond the establishment of an innate immunity to develop T_H_1 cytotoxic response against leukemia antigen-expressing cells (Ridnour et al., 2013). As our cases demonstrate, the measures needed to achieve a molecular complete response consisted of a much-shortened total treatment duration of only few weeks. This coordinated interlock of cytotoxic therapy and severe infection resembles biomodulatory therapy approaches. They aim at redirecting tumor-related pathologic homeostatic pathways (Heudobler et al., 2019). Since ALL-therapy regimens are currently being mixed up by the introduction of new drugs like monoclonal antibodies, bispecific antibodies and tyrosine kinase inhibitors (Raff et al., 2007; Paul et al., 2016), combined modality biomodulatory approaches should also be pursued. This might ultimately result in substantially shorter, less toxic therapy regimens by targeting leukemia and therapy-related stage dependent leukemia-stroma-based communicative interactions, as suggested by the two cases (Heudobler et al., 2019).

## Data Availability

The data analyzed in this study is subject to the following licenses/restrictions: The datasets generated for this study are available on request to the corresponding author. Requests to access these datasets should be directed to daniel.heudobler@ukr.de
